# Beyond technology acceptance: AI-application readiness and continuance intention among Chinese university students—a measurement-aware secondary analysis

**DOI:** 10.3389/fpsyg.2026.1963259

**Published:** 2026-10-01

**Authors:** Yunxia Sun, Yan Li

**Affiliations:** College of Media Technology, Liaocheng University, Liaocheng, China

**Keywords:** artificial intelligence, China, continuance intention, discriminant validity, higher education, secondary data, student support, technology acceptance

## Abstract

**Purpose:**

This measurement-aware secondary analysis examines whether five theoretically distinct AI-acceptance and continuance domains are empirically separable in 301 records from a public dataset described by its repository as university-student responses from mainland China.

**Method:**

The analysis used 301 records in a public workbook described by its repository as responses from university students in mainland China. Five 4-item questionnaire blocks were reconstructed from the public workbook. The measurement audit combined internal-consistency assessment, HTMT, principal-component analysis, one- versus five-factor CFA benchmarks, and a collapsed descriptive 16-item summary score. HC3-robust regressions were retained as descriptive composite benchmarks, while bootstrap and fsQCA analyses were treated as supplementary diagnostics. Perceived practical usefulness refers to respondents’ perceived career, efficiency, competitiveness, and problem-solving value rather than verified learning, employment, or institutional outcomes.

**Results:**

The five domains were internally reliable but not empirically distinct. HTMT estimates ranged from 0.983 to 1.007, the first unrotated component explained 58.75% of item variance, and the five-factor CFA provided negligible improvement over the one-factor model, with latent correlations ranging from 0.980 to 0.997. A collapsed 16-item descriptive composite was strongly associated with continuance intention (*β* = 0.896, *R*^2^ = 0.805). The regression coefficients therefore describe how shared variance is distributed across the questionnaire composites rather than independent acceptance mechanisms. fsQCA produced one high-readiness configuration and its symmetric low-readiness counterpart, with no evidence of equifinality or configurational asymmetry.

**Contribution:**

The study provides a reproducible measurement critique of a public AI-acceptance dataset. Its central finding is inadequate discriminant separation among the five affirmative questionnaire blocks. Their shared covariance can be summarized descriptively, but the present data cannot determine whether that common dimension is substantive, method-related, or both, and it should not be treated as a newly validated AI-readiness construct.

## 1 Introduction

Artificial intelligence has become part of the ordinary infrastructure of higher education. Students use general-purpose and domain-specific systems to search for information, explain unfamiliar material, generate alternatives, organize ideas, automate routine steps, receive feedback, and prepare for work that increasingly involves human-AI collaboration. The institutional question is consequently no longer whether students encounter AI, but how their encounters become sustained, academically defensible, and educationally useful. Reviews of AI in higher education describe extensive activity in assessment, prediction, tutoring, personalization, administrative support, and content generation, yet they also show that the evidence remains fragmented across technologies, disciplines, and outcome definitions (Bond et al., 2024; Crompton and Burke, 2023; Ouyang et al., 2022). Broader reviews of artificial intelligence in education similarly identify opportunities for personalization, feedback, and learner support, alongside challenges involving ethics, equity, educator preparedness, and data governance (Chiu et al., 2023). The rapid spread of generative AI has amplified this fragmentation because a single platform can function as a tutor, writing aid, search interface, coding assistant, planning tool, or shortcut, depending on the student’s purpose and judgment.

The educational value of AI therefore depends on how it is used. A system may be available but unused, used frequently but poorly integrated, or valued for convenience rather than for supporting sustained learning. Reported benefits include rapid explanations, idea generation, personalized assistance, and easier task completion, but students also have concerns about inaccuracy, privacy, dependence, authorship, and loss of autonomy (Baek et al., 2024; Chan and Hu, 2023; Grassini, 2023). These mixed evaluations demonstrate that positive educational outcomes do not follow automatically from access to AI. Results from experiments and synthesis studies suggest that including AI-powered learning can have a positive impact on some performance, affective, and higher-order learning outcomes, though the impact size differs based on the context, duration, instructional design, and level of human guidance (Deng et al., 2025; Luo et al., 2025). Any credible study of application pathways therefore needs to link technology appraisal to the behaviors in which students plan to persist using AI.

Much of the technology-adoption literature builds on the Technology Acceptance Model, which proposes that perceived usefulness and perceived ease of use shape behavioral intention (Davis, 1989). This provides an appropriate theoretical starting point, but behavioral intention alone is an incomplete educational endpoint. More recent higher-education studies have extended acceptance models by incorporating satisfaction, habit, social influence, AI competence, and facilitating conditions. These studies show that students’ intentions to use AI reflect both perceived value and the effort required to engage with the technology (Delcker et al., 2024; Strzelecki, 2024; Yu et al., 2024). Conversely, outcome-based studies examine engagement, self-regulation, creative output, learning outcomes, or career readiness, but they do not always explain how favorable appraisals translate into continued use (Zimmerman, 2002). These two literatures therefore remain adjacent but insufficiently integrated.

This disconnect is particularly important for student-affairs staff, learning-support units, academic advisors, and college counselors. Their responsibilities are not confined to a single discipline. They support students with different levels of confidence, access to resources, career goals, ethical awareness, cultural experience, and risk of overreliance. Evidence drawn only from a specific English-writing or engineering module provides limited guidance for this institution-wide role. The practical question is therefore which combination of perceived usability, usefulness, learning orientation, and intention characterizes students who expect to continue using AI in study or work. Existing policy frameworks appropriately emphasize governance, academic integrity, privacy, and AI literacy, but they provide less evidence about the student-level pathway to continued application (Chan, 2023; Cotton et al., 2024; UNESCO, 2023).

China provides an important context for this question. Students at universities in mainland China may encounter a rapidly changing platform ecology that includes domestic and international AI systems, while also facing pressures related to digital competence, learning efficiency, academic integrity, and employment preparation. These contextual observations do not imply that all Chinese university students share the same experiences. Recent Chinese evidence shows variation in perceptions of personalized AI learning environments, strategic self-regulated learning with AI, and perceived gains in efficiency, initiative, and creativity (Bai and Wang, 2025; Fan et al., 2025; Liu et al., 2025; Wang et al., 2024). However, much of this evidence is discipline-specific, context-specific, or based on instruments that do not connect general usability, usefulness, learning attitude, initial intention, and continuance in one model.

Four gaps follow from this literature. The theoretical gap is that acceptance and practical-impact perspectives are commonly separated: intention is modeled without downstream continuation, or benefits are discussed without an adoption mechanism. The empirical gap is the limited evidence from cross-disciplinary Chinese university samples using general, non-language-specific AI measures. The methodological gap is twofold. First, most studies estimate average partial associations without testing whether a conjunction of conditions is associated with continued use. Second, measurement overlap is often treated as a technical inconvenience rather than a substantive limit on what a structural model can claim. The contextual gap concerns student-support practice: research frequently addresses instructors or course design, while implications for counselors and institution-wide support remain underdeveloped.

This study conducts a measurement-aware secondary analysis of the public dataset Factors Influencing Chinese University Students’ Learning Attitudes and Intentions for Continuous Use of Artificial Intelligence Courses (Kuo and Chen, 2025). The workbook contains 301 complete responses and five 4-item blocks covering perceived practical usefulness, perceived ease of use, learning attitude, intention to use, and continuance intention. The primary question is not whether a five-construct TAM–continuance mechanism can be confirmed, but whether these affirmative domains remain empirically distinguishable when their measurement structure is examined directly.

This measurement question is psychologically consequential rather than merely technical. Acceptance models assume that students can respond to ease of use, usefulness, attitude, intention, and continuance as sufficiently differentiated evaluative dimensions for construct-specific mechanisms to be interpreted. If responses to these domains move almost interchangeably, a pathway such as ease → usefulness → attitude may partly represent decomposition of a global evaluation rather than a sequence of distinct psychological processes. The present pattern is therefore consistent with the possibility that respondents approached the affirmative AI items through a broad global impression. Because the same pattern could also arise from common-method or response-style variance, this interpretation is treated as a psychological hypothesis to be tested in future instruments rather than as a demonstrated cognitive characteristic of the respondents.

Three research questions guide the analysis.

*RQ1*: Do reliability, HTMT, PCA, CFA, and composite-correlation diagnostics support empirical separation of the five questionnaire domains?

*RQ2*: How strongly is a collapsed descriptive summary of the four pre-continuance blocks associated with continuance intention?

*RQ3*: When conventional path, coefficient-product, and configurational analyses are applied as descriptive diagnostics, do they reveal information beyond the dominant shared covariance identified by the measurement audit?

The contribution of this study is deliberately bounded to transparent secondary analysis, reproducible measurement auditing, and interpretation of the dataset’s empirical limits. When the domains fail discriminant-validity assessment, the regression and bootstrap results are retained only as descriptive composite decompositions and as a benchmark for future users of the dataset. Perceived practical impacts refer to respondents’ reported career, efficiency, competitiveness, and problem-solving value and not to objectively verified outcomes.

## 2 Theoretical framework

### 2.1 From access to sustained application

AI adoption in higher education is sometimes discussed as a binary event: students either use a tool or they do not. This framing conceals meaningful variation. Students can experiment once, use AI intermittently, integrate it into recurring study routines, or continue developing AI-related knowledge for future work. Continued application is therefore a more demanding state than initial exposure.

Bhattacherjee’s (2001) expectation-confirmation logic distinguishes acceptance from continuance by arguing that post-adoption evaluation shapes whether technology use persists. Although the present dataset does not measure confirmation or satisfaction directly, it contains the core sequence of perceived value, attitude, intention, and continued use. This permits an examination of whether favorable evaluation is linked to a sustained application orientation rather than a one-time decision.

This separation is supported by recent evidence. Satisfaction and perceived value are important correlates of continued ChatGPT use (Yu et al., 2024), and for university students, usefulness, effort expectancy, habit, and related acceptance factors have been identified as correlates of students’ adoption intentions (Strzelecki, 2024). Delcker et al. (2024) also showed that, in the case of first-year students, AI competence and positive attitudes contribute to understanding the intended and actual use. These studies share a fundamental premise: continued use requires more than technology availability. It depends on students’ ability to use the system, recognition of value in repeated use, and a sufficiently favorable evaluation of continued engagement.

Continued use, however, is not inherently desirable. A student may continue using AI because it supports planning, feedback, or problem solving, but continued use may also involve uncritical outsourcing of academic work. Research on academic integrity highlights the potential for generative AI to obscure authorship and decrease visibility of the process (Cotton et al., 2024; Perkins, 2023). Reviews also identify hallucination, bias, privacy risks, and dependency as persistent concerns (Farrokhnia et al., 2024; Kasneci et al., 2023; Tlili et al., 2023). Therefore, the present study treats continuance intention as an application-related outcome rather than an indicator of educational quality. Its educational value depends on the practical and ethical context of use.

Continuance intention should be distinguished from frequency, intensity, and quality of AI use. The present dataset captures stated continuation, but it does not establish whether use is augmentative, ethically disclosed, critically evaluated, or educationally effective. These dimensions require behavioral, task-based, and longitudinal evidence and therefore remain outside the present analysis.

### 2.2 Perceived ease of use and practical usefulness

TAM proposes that attitudes toward technology and behavioral intentions are influenced by perceived ease of use and perceived usefulness (Davis, 1989). Perceived ease of use refers to the effort required to learn and operate a system. In AI-supported learning contexts, ease of use involves a clear interface, low learning costs, intuitive operation, and sufficient familiarity to support self-directed engagement. Greater ease of use may reduce avoidable friction and allow students to focus more attention on evaluating AI outputs and applying them in academic or career-related activities. However, ease is ambivalent in terms of education. An unobtrusive interface can aid in deliberate investigation or in copying unconsciously. Ease of use is therefore best treated as an enabling condition whose educational significance depends on the value and quality of the resulting use.

The practical-usefulness items extend beyond immediate academic performance. These items ask whether learning about AI may support future career development, work productivity, competitiveness, and everyday problem solving. These are “practical usefulness” indicators: the beliefs that AI competence is useful in other contexts, such as study, work, and life. The student surveys indicate that efficiency, personalized support, and employability are significant drivers for the use of AI (Baek et al., 2024; Chan and Hu, 2023), confirming this interpretation. This interpretation is also consistent with Chinese studies in which students associate AI with initiative, information access, and creative support while remaining concerned about output reliability (Fan et al., 2025; Wang et al., 2024).

Theoretically there is a link between ease and usefulness, but they are not equivalent. A user-friendly system may nevertheless be perceived as irrelevant, whereas a valuable system may require substantial effort. Lower required effort may strengthen perceived usefulness by making the system’s benefits easier to access. The two item blocks in the present data, however, could be representative of a general positive attitude toward AI. The first relationship is therefore examined as an exploratory proposition requiring discriminant-validity assessment: EP1, perceived ease of use is positively associated with perceived practical usefulness.

### 2.3 Learning attitude as an evaluative bridge

Learning attitude encompasses interest, enjoyment, perceived value, and a positive orientation toward continued AI-supported learning. It appears to be a middle ground between technology appraisal and behavioral intention. Students may regard AI as useful without enjoying learning with it, or may enjoy learning about AI without believing that it supports their longer-term goals. Attitude gathers these evaluations together by integrating whether learning with AI is perceived as being valuable and emotionally acceptable.

This distinction is important because higher-education research reports both enthusiasm for AI-supported learning and caution regarding its limitations. Students often value personalization and speed while remaining uncertain about reliability, authenticity, and the risk of overdependence (Chan and Hu, 2023). Additionally, there are differences in judgments between faculty and students about ease, enjoyment, habitual use, and appropriate boundaries (Kim et al., 2025). When AI is manageable and useful, it is reasonable to expect a positive attitude, but by no means is it a blanket endorsement. Perceived value may provide a particularly strong evaluative bridge when accompanied by usability.

This framework suggests two exploratory propositions. EP2a: perceived ease of use is positively associated with learning attitude. EP2b: perceived practical usefulness is positively associated with learning attitude. The joint equation tests whether each appraisal remains associated with attitude when the other appraisal is held constant. Given the high conceptual proximity of the scales, the analysis also reports variance inflation factors and discriminant diagnostics rather than treating the coefficients as independent structural mechanisms.

### 2.4 Intention and continuance intention

Behavioral intention captures a student’s willingness to use AI repeatedly, prioritize AI-supported learning, devote additional time to learning about AI, and recommend it to others. TAM is based on the assumption that intention is the first step toward use. In the current framework, learning attitude is assumed to be a variable that may be correlated to intention due to interest, enjoyment, and perceived value, as they decrease resistance to conscious use. A direct association is also plausible because students may intend to use AI for instrumental reasons even when their affective response is less favorable.

These relationships are in line with recent research in higher education. Both Strzelecki (2024) and Yu et al. (2024) demonstrate that perceptions of performance and satisfaction are associated with intention and continuation. Positive attitudes toward AI are tied to the intended and actual use of AI (Delcker et al., 2024), and the usage patterns vary between academic and demographic groups (Stöhr et al., 2024). Accordingly, two exploratory propositions are examined: EP3a, learning attitude is positively associated with intention to use AI; and EP3b, perceived practical usefulness is positively associated with intention to use AI.

Continuance intention is the final outcome. Its indicators involve continued AI learning after a course, application of AI knowledge to study or work, attention to new developments, and participation in communities or forums. This is broader than repeated use of a particular chatbot because it captures a sustained orientation toward AI capability and application. Expectation-confirmation theory would normally include satisfaction and confirmation as post-use mechanisms (Bhattacherjee, 2001); those measures are absent here. Within the available indicators, intention, attitude, and practical usefulness are the most defensible direct antecedents. Accordingly, three exploratory propositions are examined: EP4a, intention to use AI is positively associated with continuance intention; EP4b, learning attitude is positively associated with continuance intention; and EP4c, perceived practical usefulness is positively associated with continuance intention.

### 2.5 Indirect pathways

The sequential logic implies several indirect associations. Ease may be linked to continuation through usefulness because a clear, low-cost system allows students to recognize practical benefits. It may also operate through attitude, through the usefulness-attitude sequence, or through longer sequences that culminate in intention. Likewise, usefulness may be linked to continuation through attitude, intention, or both. These pathways should not be described as causal mediation because the data are cross-sectional and temporal order was not observed. They are coefficient-product decompositions that evaluate whether the proposed sequence is statistically coherent.

The indirect relationships are examined as exploratory coefficient-product propositions. EP5a concerns positive indirect associations between perceived ease of use and continuance intention through practical usefulness and/or learning attitude. EP5b concerns positive serial indirect associations from ease through usefulness, attitude, and intention. EP5c concerns positive indirect associations from practical usefulness through attitude and/or intention. EP5d concerns a positive indirect association from learning attitude through intention. Bootstrap confidence intervals are used because coefficient-product distributions are rarely normal.

### 2.6 Configurational reasoning and measurement-aware theory

Regression estimates the average partial association of each predictor while holding the other predictors constant. It does not test whether combinations of conditions are sufficient for a high outcome. In set-theoretic analysis, each case is represented as a configuration, allowing examination of conjunction, equifinality, and asymmetry (Fiss, 2011; Ragin, 2008). Pappas and Woodside (2021) argue that fsQCA can complement variance-based methods when information-systems outcomes depend on bundles of conditions. In the present context, continued AI application may require the joint presence of usability, usefulness, positive attitude, and intention, or some conditions may compensate for others. These possibilities are evaluated rather than assumed.

Related work in Chinese higher education has shown that configurational analysis can reveal multiple sufficient combinations when the underlying conditions retain meaningful heterogeneity. For example, Zheng et al. (2023) combined SEM and fsQCA to examine Chinese college students’ e-learning continuance, while Zhou and Zhang (2024) used SEM and fsQCA to examine continuance intentions toward AI tools for self-directed learning among university students in China. Against that evidence, the lack of equifinality in the present data is informative primarily as a diagnostic consequence of the unusually strong overlap among the conditions rather than as evidence of multiple independent configurational mechanisms.

Given the extreme overlap among the four conditions, fsQCA is not used here on the assumption that multiple configurational recipes must exist. It is used as a diagnostic test of configurational diversity. Multiple non-symmetric sufficient configurations would indicate heterogeneity that is not captured by a single readiness score. Conversely, one symmetric high/low solution with no equifinality or asymmetry would be descriptively consistent with a dominant shared-response gradient, while not constituting independent evidence for a distinct readiness mechanism.

The configurational proposition is therefore open-ended: EP6, one or more configurations of perceived ease, practical usefulness, learning attitude, and intention will be sufficient for high continuance intention, and the configuration for low continuance need not be the simple inverse of the high-outcome solution. Testing this proposition is useful even if equifinality is not found. A single conjunctural solution would indicate that the sample behaves more like an overall readiness gradient than a set of substitutable pathways.

A second theoretical issue concerns measurement. Structural models often proceed from acceptable alpha values to claims about distinct constructs. Reliability, however, is not discriminant validity. High interconstruct correlations and HTMT values near one indicate that respondents may not distinguish ease, usefulness, attitude, intention, and continuation as sharply as theory proposes. Hair et al. (2022) recommend evaluating reliability and validity together; reliable but non-discriminant blocks cannot support strong latent-variable interpretation. The present study treats this possibility as a central test. If the item blocks collapse into a broad favorable AI orientation, the practical contribution shifts from identifying precise causal mechanisms to describing a coherent readiness profile and designing better future measurement.

The measurement question also bears on theory portability. Constructs developed for conventional information systems are often adapted to generative AI by changing the name of the technology while retaining highly affirmative wording. Generative AI, however, combines tool usability, conversational interaction, epistemic uncertainty, and authorship risk. Research on student agency further suggests that AI use can range from surface-level task completion to deeper learning, depending on how students frame, evaluate, and integrate generated outputs (Yang et al., 2024). Students may answer a group of positive items according to a global impression rather than making fine distinctions among effort, value, enjoyment, intention, and continuation. A more discriminating instrument would include context-specific scenarios, negatively valenced or caution-oriented items, and indicators that separate instrumental benefit from affective enthusiasm. It would also distinguish use of AI from learning about AI. In the present workbook, several items refer to AI learning systems or courses, while others refer to applying AI knowledge in study or work. This breadth supports a general higher-education interpretation, but it also increases conceptual blending. The article therefore treats measurement design as part of the substantive explanation of why the observed pathway appears unusually strong.

Three competing interpretations are considered. The first is the proposed sequential ordering: students who perceive AI systems as easier to use may judge them as more useful; usefulness and ease may accompany a more favorable learning attitude; and attitude and usefulness may accompany intention and continuance. The second is reciprocal appraisal: students who already intend to continue using AI may retrospectively rate all preceding domains more favorably. The third is a common-orientation interpretation in which all five blocks reflect a broad favorable attitude toward AI learning and application. Because the survey is cross-sectional, it cannot establish temporal direction between the first two interpretations, while the HTMT, PCA, CFA, and collapsed-readiness results provide substantial support for the third. These alternatives would require longitudinal, cross-lagged, experimental, or behavioral designs to distinguish. Figure 1 therefore represents an exploratory analytical ordering rather than a confirmed causal or temporal model.

**Figure 1:**
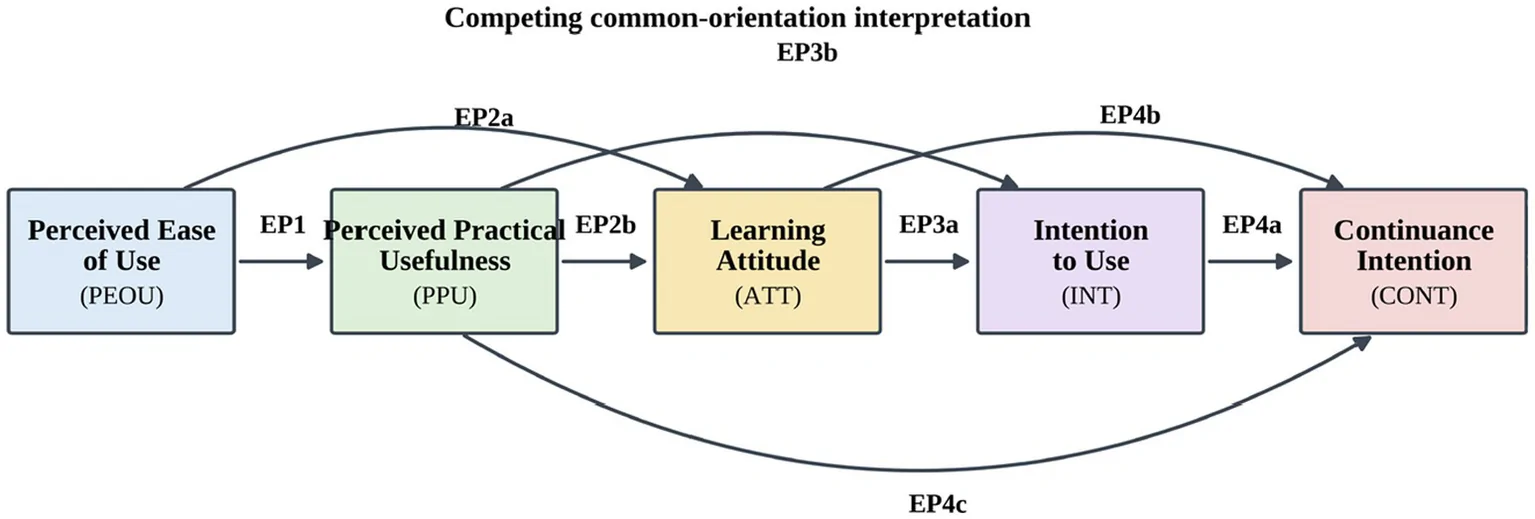
Exploratory analytical ordering of the five AI-appraisal questionnaire composites. Arrows represent prespecified composite associations rather than causal or temporally verified mechanisms. The dashed boundary represents a competing shared-orientation interpretation that may contain substantive and/or method-related common variance.

## 3 Method

*Analytical positioning*: The path regressions, bootstrap coefficient-product decompositions, and fsQCA are retained as diagnostic demonstrations of what a conventional TAM-style analysis would appear to show if the severe measurement overlap were ignored. They are not confirmatory tests of five distinct mechanisms. Their purpose is to demonstrate that statistically significant-looking paths, indirect products, and configurations can arise largely from shared variance among the affirmative questionnaire blocks. Accordingly, all coefficients, coefficient products, and set-theoretic solutions are interpreted as descriptive decompositions of a general AI-application readiness profile, not as causal, temporal, or construct-specific effects.

### 3.1 Research design and data source

This study used a quantitative, cross-sectional secondary-analysis design. The source is a public Mendeley Data package titled Factors Influencing Chinese University Students’ Learning Attitudes and Intentions for Continuous Use of Artificial Intelligence Courses, version 1 (Kuo and Chen, 2025) licensed under CC BY 4.0. The repository provides a description of 301 valid questionnaires from students at universities in mainland China for various academic levels and disciplines. This analysis was performed on a raw workbook (0308AI.xlsx) that is included with the survey. No additional participants were recruited and no “direct identifiers” were accessed.

An internal integrity audit of the downloaded workbook confirmed 301 respondent rows in the primary worksheet and a second worksheet containing the same 20 focal item responses for all 301 records. No additional worksheet providing demographic category labels, institution identifiers, regional identifiers, or a sampling frame was present. The workbook therefore permits verification of the internal response matrix but not independent reconstruction of recruitment, institutional coverage, regional coverage, or the substantive meanings of all coded demographic categories. The analyzed file is consequently treated as a public secondary dataset whose respondent provenance is bounded by the documentation supplied with the repository. During revision, the dataset authors were contacted to request clarification of respondent recruitment, participating institutions and regions, category coding, timestamp generation, and the relationship between the public workbook and Chen et al. (2025). No response had been received by the time the revised manuscript was finalized. Consequently, these aspects remain unverified beyond the public repository documentation. The workbook-level integrity checks and the status of the external clarification attempt are summarized in Supplementary Table S1.

### 3.2 Differentiation from the dataset authors’ prior publication

The public dataset is associated with a related conference publication by members of the dataset-author team (Chen et al., 2025). Table 1 distinguishes the research focus, variables, analytical purpose, and reported outputs of that publication from the present secondary analysis. Chen et al. (2025) is a formally published ACM conference-proceedings article associated with the same public data resource, rather than an unpublished data description. The present secondary analysis uses the public workbook as a shared data source but addresses a different measurement question and reports independently generated reliability, discriminant-validity, PCA, CFA, collapsed-score, robust-regression, bootstrap, and configurational outputs. No numerical estimate, table, figure, or inferential conclusion from Chen et al. (2025) is reproduced as an original result of the present study.

**Table 1:** Differentiation between Chen et al. (2025) and the present secondary analysis.

Dimension	Chen et al. (2025)	Present study
Primary research focus	Relationships among AI-use attitude, AI-use behavior, learning motivation, and learning satisfaction	Whether five apparently distinct acceptance and continuance domains are empirically separable or collapse into a general AI-application readiness profile
Focal variables	AI-use attitude, AI-use behavior, learning motivation, and learning satisfaction	PPU, PEOU, ATT, INT, CONT, a 16-item general-readiness composite, and coded demographic and usage controls
Analytical approach	Original relationship analysis of the four focal variables as reported in the conference article	Reliability and discriminant-validity audit; HTMT; PCA; one-factor versus five-factor CFA benchmarks; HC3-robust descriptive composite regressions; 10,000-draw bootstrap coefficient-product decomposition; collapsed-readiness regression; and fsQCA
Primary analytical purpose	Substantive examination of motivation and satisfaction in relation to AI-use attitude and behavior	Measurement critique, reproducible secondary analysis, and evaluation of whether the dataset supports domain-specific interpretation
Results reproduced from prior publication	Not applicable	Not applicable
Distinct contribution of present study	Not applicable	Demonstration that the five affirmative blocks have severe empirical overlap and are more defensibly interpreted as a general readiness profile

No numerical result, table, figure, model estimate, or inferential conclusion from Chen et al. (2025) is duplicated in the present article. All statistics reported here were generated independently from the public workbook using the accompanying analysis code.

### 3.3 Unit of analysis, sample, and scope

Respondent records are the unit of analysis. The analytical sample comprised 301 records contained in the public workbook. The workbook includes coded fields for gender, age, study stage, department, primary purpose of the use of AI, and frequency of use of AI. There is no public codebook with substantive labels for all numeric categories. For this reason, the present research does not generate categories, labels or frequencies like specific age bands, departments, or usage reasons. The same code is used for the AI-use screening item for all observations and it is not used as a predictor.

Each of the 20 scale items has a score of 1–5. There are no missing item values. The data are said to be mainland Chinese and multi-university, but information on institutions, province-level sampling fractions and a probability sampling frame are not provided. Accordingly, the findings describe the surveyed students and should not be assumed to represent Chinese higher education broadly.

More specifically, the substantive conclusions are restricted to the 301 records contained in the analyzed public workbook. References to the mainland Chinese university context indicate the population description supplied by the repository and should not be interpreted as population-level estimates for Chinese university students as a whole.

### 3.4 Variable operationalization

Five composite scores were reconstructed as unweighted means of their respective questionnaire items. Beliefs regarding future career development, work efficiency, competitiveness, and problem solving are included in perceived practical usefulness (PPU). Perceived ease of use (PEOU) refers to clarity, low learning cost, ease of self-learning, and ease of operation. Learning attitude (ATT) involves interest, pleasure, the perceived value of the investment, and a positive attitude toward ongoing learning in AI. Intention to use (INT) captures respondents’ stated willingness to use AI repeatedly, prioritize AI-supported learning, spend additional time learning about AI, and recommend AI courses to others. Continuance intention (CONT) captures continued AI learning after a course, application of AI knowledge in study or work, monitoring of new AI developments, and participation in AI-related communities or forums.

PPU is not an objective impact measure; it represents perceived prospective practical value. CONT is not observed long-term behavior; it represents stated continuance intention. These distinctions are maintained in the title interpretation, abstract, results, and conclusion. The demographic and usage variables are entered as coded controls through dummy variables, with the lowest observed code used as the reference category. Because the public workbook does not provide a codebook, the control coefficients are neither assigned substantive labels nor interpreted in the main text.

### 3.5 Data-quality and measurement assessment

Data-quality checks covered missingness, item ranges, exact duplicate numeric profiles, straight-lined responses across the 20 focal items, maximum identical-response runs, and response time. All focal items fell within the expected 1–5 range. Response-time values were extracted from the workbook. Submission timestamps were compressed into an interval of approximately 13 min. This pattern may reflect batch export, anonymization, synchronized administration, or another data-processing artifact; the public materials do not permit a definitive explanation. It is therefore reported as a provenance limitation rather than as evidence that the responses are invalid.

Cronbach’s alpha was used to assess the internal consistency. Standardized item loadings, composite reliability and average variance extracted were obtained for each block. Correlations and HTMT were computed for inter-construct relationships. HTMT for domains *a* and *b* was calculated as the mean absolute correlation between items assigned to different domains divided by the geometric mean of the corresponding within-domain absolute item correlations:

$${\text{HTMT}}_{\mathrm{ab}}=\frac{\frac{1}{K_{a}K_{b}}\sum_{i=1}^{K_{a}}\sum_{j=1}^{K_{b}}|r_{\mathrm{ij}}|}{\sqrt{\left[\frac{2}{K_{a}(K_{a}-1)}\sum_{i<j}|r_{\mathrm{ij}}^{\left(a\right)}|][\frac{2}{K_{b}(K_{b}-1)}\sum_{i<j}|r_{\mathrm{ij}}^{\left(b\right)}|\right]}}$$

Pearson item correlations were used; the correlation operation standardizes the item scores internally. Percentile 95% confidence intervals were obtained from 10,000 case-resampling bootstrap samples using random seed 20,260,728 (Henseler et al., 2015).

A general common factor diagnostic was an unrotated principal-component analysis of all 20 items. A Harman-type first-component diagnostic cannot establish the presence or absence of common-method bias; it was interpreted only alongside the HTMT, PCA, and CFA evidence. Because discriminant validity was questionable, the analysis did not proceed to confirmatory latent structural modeling. A confirmatory factor-analysis benchmark compared a correlated five-factor measurement model with a one-factor model using normal-theory maximum-likelihood covariance estimation. Latent variances were fixed to one for identification, each item loaded only on its pre-specified factor in the five-factor model, and residual variances were freely estimated. Model fit was evaluated using chi-square, the comparative fit index (CFI), Tucker–Lewis index (TLI), root mean square error of approximation (RMSEA), and standardized root mean square residual (SRMR). The CFA was used only as a measurement diagnostic and not to justify a latent structural model.

The measurement assessment proceeded in three stages: response-pattern screening, within-block reliability assessment, and discriminant-validity assessment. Failure of the third stage did not preclude descriptive composite analysis, but it precluded interpreting coefficient differences as relations among distinct latent mechanisms. The pre-specified decision rule was to retain the regression equations only as descriptive benchmarks when HTMT values exceeded conventional thresholds or a dominant general factor was evident.

Model specification was determined *a priori* from the questionnaire order and TAM/continuance theory. No data-driven predictor selection, coefficient-based deletion, or threshold optimization was performed. The fsQCA calibration anchors and truth-table thresholds were fixed before solution evaluation and were retained in the sensitivity analyses.

To examine whether the domain-specific results could be represented more parsimoniously, a collapsed general-readiness benchmark was also estimated. The complete 20-item score was used to assess overall internal consistency and descriptive distribution. To avoid predictor–outcome item overlap, a separate 16-item readiness composite based on PPU, PEOU, ATT, and INT was standardized and entered as the focal predictor of CONT. The regression included the same coded controls and used HC3 heteroskedasticity-consistent standard errors.

### 3.6 Associational path models

Standardized composite scores were used in four ordinary least-squares equations with HC3 heteroskedasticity-consistent standard errors. Let *C*_*i* represent the vector of coded controls for student *i*:

$${\mathrm{PPU}}_{i}=\beta_{0}+\beta_{1}{\text{PEOU}}_{i}+\Gamma C_{i}+\varepsilon_{1i}$$

$${\mathrm{ATT}}_{i}=\alpha_{0}+\alpha_{1}{\text{PEOU}}_{i}+\alpha_{2}{\mathrm{PPU}}_{i}+\Lambda C_{i}+\varepsilon_{2i}$$

$${\mathrm{INT}}_{i}=\gamma_{0}+\gamma_{1}{\mathrm{ATT}}_{i}+\gamma_{2}{\mathrm{PPU}}_{i}+\Phi C_{i}+\varepsilon_{3i}$$

$${\text{CONT}}_{i}=\delta_{0}+\delta_{1}{\mathrm{INT}}_{i}+\delta_{2}{\mathrm{ATT}}_{i}+\delta_{3}{\mathrm{PPU}}_{i}+\Omega C_{i}+\varepsilon_{4i}$$

All focal variables were standardized before estimation, so focal coefficients are standardized composite associations. HC3 heteroskedasticity-consistent standard errors were used because they provide improved finite-sample protection against leverage-sensitive heteroskedasticity (MacKinnon and White, 1985). Controls were included to reduce simple demographic and usage confounding, but their coded categories are not interpreted. Variance inflation factors were calculated for focal predictors. Because the measurement audit indicated severe overlap, these equations are not presented as a validated latent SEM.

### 3.7 Bootstrap coefficient-product associations

Indirect associations were computed as products of standardized path coefficients. For example, the indirect association PEOU → PPU → CONT is represented by $\beta_{1}\delta_{3}$ , whereas the longer sequence PEOU → PPU → ATT → INT → CONT is represented by $\beta_{1}\alpha_{2}\gamma_{1}\delta_{1}$ . Percentile confidence intervals were obtained from 10,000 case-resampling bootstrap samples with a fixed random seed of 20,260,728. Controls were resampled with cases. A bootstrap interval excluding zero was interpreted as support for an indirect association, not proof of temporal mediation. Product terms were evaluated by resampling because their sampling distributions are generally asymmetric and poorly approximated by normal-theory tests (MacKinnon et al., 2004).

### 3.8 Fuzzy-set qualitative comparative analysis

fsQCA was used to examine whether combinations of PPU, PEOU, ATT, and INT were sufficient for high or low CONT (Schneider and Wagemann, 2012). Each five-point composite was calibrated through a direct logistic function using 1.5 as full non-membership, 3.0 as the crossover, and 4.5 as full membership. The crossover of 3.0 corresponds to the midpoint of the five-point response scale. The symmetric 1.5 and 4.5 anchors were selected to require responses to lie clearly toward the lower or upper end of the scale before approaching full non-membership or membership, rather than assigning near-complete set membership only to exact scores of 1 or 5. These anchors also lie well within the observed composite distributions, whose means ranged from 3.239 to 3.286 and whose standard deviations ranged from 1.103 to 1.131. To evaluate calibration dependence, the analysis was repeated using the scale endpoints 1.0 and 5.0 with the same 3.0 crossover. The same mirror-image high- and low-outcome configurations were retained. Under the 1.0/3.0/5.0 calibration, the high-CONT configuration had consistency = 0.965, PRI = 0.944, and raw coverage = 0.796; the low-CONT configuration had consistency = 0.959, PRI = 0.930, and raw coverage = 0.720.

Exact 0.5 scores were adjusted minimally to avoid ambiguous set allocation. Necessity was screened before sufficiency. The truth table required at least five cases, consistency of 0.80, and proportional reduction in inconsistency (PRI) of 0.65. Complex solutions were retained because they do not simplify with unobserved logical remainders. Sensitivity checks raised consistency to 0.85 and PRI to 0.70. For condition configuration *X* and outcome *Y*, sufficiency consistency was computed as sum min (*X*_*i*, *Y*_*i*)/sum *X*_*i*, and raw coverage as sum min (*X*_*i*, *Y*_*i*)/sum *Y*_*i*. Negated conditions are denoted by ~ and conjunction by multiplication. fsQCA was used as a diagnostic of configurational diversity: multiple asymmetric recipes would indicate heterogeneity beyond a single readiness gradient, whereas one symmetric high/low solution would support the general-readiness interpretation.

### 3.9 Reproducibility and ethics

All analyses were performed in Python. The accompanying script imports the public workbook, reconstructs the five composites, performs the data-quality checks, calculates block-level reliability, HTMT, PCA, the one-factor and correlated five-factor CFA benchmarks, and the collapsed general-readiness model, estimates the HC3-robust composite regressions, computes 10,000 case-resampling bootstrap indirect associations, conducts fsQCA and sensitivity analyses, and exports machine-readable tables, figures, and a JSON summary.

This study involves secondary analysis of previously collected, de-identified human-subject data. No new participants were recruited, and no direct identifiers were accessed. Because only publicly available anonymized secondary data were analyzed, no new ethics approval was sought for the present secondary analysis.

## 4 Results and discussion

### 4.1 Preliminary audit and sample profile

All 301 cases had complete focal-item data, and every item fell within the expected 1–5 range. No exact duplicate numeric profiles or straight-lined 20-item response profiles were identified. The longest identical-response run across the 20 focal items was eight items. Mean response time was 173.4 s and median response time was 164 s. These checks do not validate the sampling process or explain the compressed timestamps, but they reduce concern about obvious duplication and universal straight-lining.

The timestamp interval was 13 min 11 s, from 14:51:09 to 15:04:20, with 184 unique submission times among the 301 records. Recorded response durations ranged from 91 to 281 s (*M* = 173.4, median = 164). The 20 focal items contained no exact duplicate response profiles and no complete straight-lined profiles. As a sensitivity check, the collapsed 16-item summary remained strongly associated with continuance intention after linear submission time and response duration were added to the coded controls (*β* = 0.887, HC3 SE = 0.030, 95% CI [0.829, 0.946], *p* < 0.001; *R*^2^ = 0.806), compared with *β* = 0.896 and *R*^2^ = 0.805 in the primary model. These checks do not authenticate the recruitment or timestamp-generation process, but they show that the principal covariance pattern is not eliminated by adjustment for the available timing metadata.

Table 2 presents the coded sample profile. Gender codes 1 and 2 included 170 and 131 cases, respectively. The largest age category was code 1 (*n* = 201); five study-stage codes, six department codes, six use-reason codes, and six frequency codes were observed. Because the public workbook provides no substantive codebook, the categories are reported only by code. Assigning substantive labels without documentation would create unsupported sample characteristics.

**Table 2:** Coded sample profile (*N* = 301).

Characteristic	Observed coded distribution
Gender	1: 170 (56.48%); 2: 131 (43.52%)
Age	1: 201 (66.78%); 2: 65 (21.59%); 3: 23 (7.64%); 4: 12 (3.99%)
Study stage	1: 74; 2: 65; 3: 68; 4: 39; 5: 55
Department	1: 92; 2: 61; 3: 40; 4: 80; 5: 21; 6: 7
Main reason for AI use	1: 91; 2: 47; 3: 64; 4: 22; 5: 60; 6: 17
AI-use frequency	1: 57; 2: 82; 3: 95; 4: 34; 5: 22; 6: 11
AI-use screening item	All 301 cases recorded code 1
Response time	Mean = 173.4 s; median = 164 s

All codes are reported as they appear in the public workbook. No substantive labels are assigned because a complete category codebook is not publicly available.

### 4.2 Reliability, descriptives, and discriminant validity

Table 3 indicates that each four-item block was internally consistent. Alpha values ranged from 0.832 to 0.848, composite reliability from 0.888 to 0.898, and AVE from 0.664 to 0.687. The item loadings were between 0.780 and 0.865. These values appear adequate when each construct is evaluated separately. The means were very close together, ranging from 3.239 to 3.286, and had standard deviations of 1.103 to 1.131.

**Table 3:** Descriptive statistics and block-level measurement quality.

Construct	Mean	SD	Alpha	CR	AVE	Loading range
PPU	3.257	1.106	0.833	0.889	0.667	0.798–0.854
PEOU	3.239	1.131	0.844	0.895	0.681	0.808–0.835
ATT	3.286	1.113	0.832	0.888	0.664	0.798–0.840
INT	3.267	1.103	0.838	0.892	0.673	0.780–0.865
CONT	3.258	1.123	0.848	0.898	0.687	0.807–0.845

PPU, perceived practical usefulness; PEOU, perceived ease of use; ATT, learning attitude; INT, intention to use; CONT, continuance intention.

Table 4 changes the interpretation of the block-level results. Composite correlations ranged from 0.826 to 0.841, and HTMT values ranged from 0.983 to 1.007, well above conventional 0.85 or 0.90 thresholds. The first unrotated component explained 58.75% of total item variance, whereas the second explained only 3.13%. Together, these findings indicate that the five reliable item blocks are not sufficiently distinct to support separate latent-construct claims; they may instead reflect a common favorable orientation toward AI learning and application.

**Table 4:** Composite correlations and HTMT discriminant-validity diagnostics.

Panel A. Composite correlations					
Construct	PPU	PEOU	ATT	INT	CONT
PPU	1.000	0.839	0.836	0.841	0.826
PEOU	0.839	1.000	0.838	0.838	0.839
ATT	0.836	0.838	1.000	0.840	0.839
INT	0.841	0.838	0.840	1.000	0.840
CONT	0.826	0.839	0.839	0.840	1.000

Panel B. HTMT estimates with bootstrap confidence intervals		
Construct pair	HTMT	95% bootstrap CI
PPU–PEOU	1.000	[0.968, 1.033]
PPU–ATT	1.004	[0.973, 1.036]
PPU–INT	1.006	[0.975, 1.040]
PPU–CONT	0.983	[0.946, 1.018]
PEOU–ATT	1.000	[0.964, 1.036]
PEOU–INT	0.997	[0.964, 1.028]
PEOU–CONT	0.992	[0.954, 1.029]
ATT–INT	1.007	[0.975, 1.040]
ATT–CONT	0.999	[0.967, 1.033]
INT–CONT	0.997	[0.966, 1.029]

*N* = 301. Panel A reports Pearson correlations among the unweighted four-item composite scores. HTMT estimates were calculated from the absolute Pearson item correlations. Percentile 95% confidence intervals were obtained from 10,000 case-resampling bootstrap draws using random seed 20,260,728. HTMT values exceeding 0.90 indicate inadequate discriminant validity. The first unrotated component explained 58.75% of total item variance. PPU, perceived practical usefulness; PEOU, perceived ease of use; ATT, learning attitude; INT, intention to use; CONT, continuance intention.

HTMT values near or above 1.00 can coexist with composite correlations of approximately 0.84 because the two statistics evaluate different aspects of the measurement structure. Composite correlations relate the means of the four-item blocks and retain residual between-person variation. HTMT compares the average cross-domain item correlations with the corresponding average within-domain item correlations. When correlations between items assigned to different domains are approximately as large as correlations among items assigned to the same domain, the HTMT ratio approaches one even though the composite scores are not perfectly correlated. The bootstrap confidence intervals, whose lower limits all exceeded 0.94, reinforce the conclusion that the five domains lack adequate discriminant separation. To triangulate the HTMT and PCA evidence, a confirmatory factor-analysis benchmark compared the pre-specified correlated five-factor model with a one-factor model. The five-factor model showed *χ*^2^ (160) = 155.152, *p* = 0.593, CFI = 1.000, TLI = 1.000, RMSEA = 0.000, and SRMR = 0.022. The one-factor model showed *χ*^2^ (170) = 157.211, *p* = 0.750, CFI = 1.000, TLI = 1.000, RMSEA = 0.000, and SRMR = 0.022. The five-factor model produced only a negligible improvement, Δ*χ*^2^ (10) = 2.059, *p* = 0.996. Moreover, estimated latent-factor correlations ranged from 0.980 to 0.997, and the factor-correlation matrix was nearly singular. The apparently excellent absolute fit therefore does not establish discriminant validity. Taken together, the HTMT, PCA, and CFA results establish strong shared covariance and inadequate discriminant separation, but they cannot identify whether that common variance represents a substantive general evaluation of AI, common-method variance, co-directional wording, response-style effects, or a mixture of these sources.

Taken together, the HTMT, PCA, and CFA results delimit the permissible interpretation of the subsequent models. The coefficients are descriptive partial decompositions among highly related composites and do not establish five empirically distinct mechanisms; the large *R*^2^ values must therefore be read as substantially reflecting shared measurement variance.

### 4.3 Collapsed descriptive shared-response benchmark

A collapsed benchmark was estimated to determine whether the domain-specific findings could be represented more parsimoniously by a general AI-readiness profile. The complete 20-item readiness score demonstrated high internal consistency (*M* = 3.261, SD = 1.040, Cronbach’s *α* = 0.963). To avoid predictor–outcome item overlap, a separate 16-item readiness composite based on PPU, PEOU, ATT, and INT was related to CONT. After inclusion of the coded controls, general readiness was strongly associated with CONT (*β* = 0.896, HC3 SE = 0.026, 95% CI [0.844, 0.947], *p* < 0.001; model *R*^2^ = 0.805). These results show that a single summary score captures much of the covariance with continuance intention in this dataset; they do not establish the validity of a new latent readiness construct or distinguish substantive common variance from method-related common variance.

### 4.4 Descriptive HC3-robust composite decompositions

Table 5 and Figure 2 report HC3-robust standardized composite associations as descriptive benchmarks under the measurement limitations established above. PEOU was strongly associated with PPU (*β* = 0.818, 95% CI [0.755, 0.882], *p* < 0.001), and the equation explained 74.2% of PPU variance. EP1 was consistent with the observed composite association. The magnitude is substantively large, but it is also consistent with the possibility that both blocks measure the same broad orientation.

**Table 5:** HC3-robust descriptive composite decomposition estimates.

Outcome	Predictor	*β*	HC3 SE	*p*	95% CI	*R*-squared
PPU	PEOU	0.818	0.032	<0.001	[0.755, 0.882]	0.742
ATT	PEOU	0.470	0.066	<0.001	[0.342, 0.599]	0.778
ATT	PPU	0.431	0.070	<0.001	[0.294, 0.569]	0.778
INT	ATT	0.446	0.053	<0.001	[0.342, 0.550]	0.783
INT	PPU	0.480	0.052	<0.001	[0.379, 0.582]	0.783
CONT	INT	0.350	0.058	<0.001	[0.236, 0.465]	0.793
CONT	ATT	0.324	0.062	<0.001	[0.202, 0.446]	0.793
CONT	PPU	0.267	0.066	<0.001	[0.137, 0.396]	0.793

HC3, heteroskedasticity-consistent covariance estimator type 3. Coefficients are standardized composite associations and should not be interpreted as causal effects.

**Figure 2:**
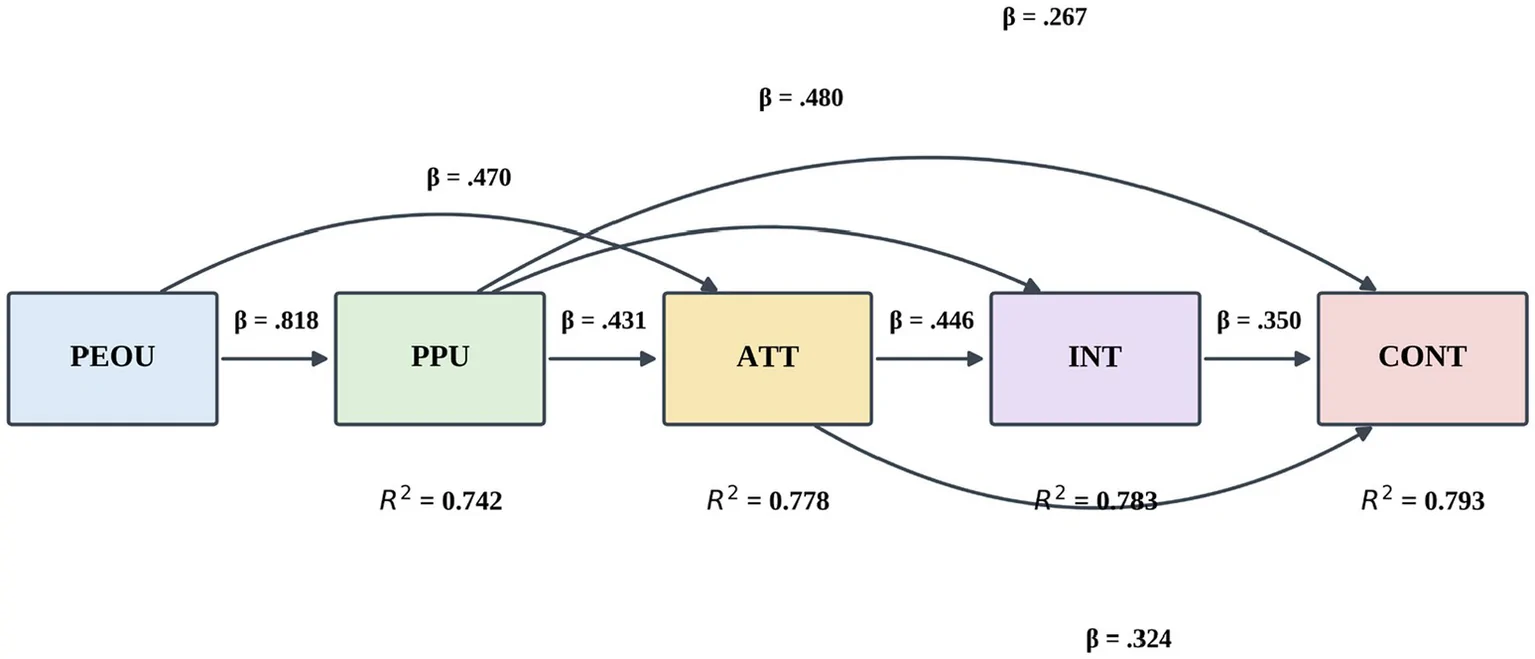
HC3-robust standardized composite associations among the questionnaire composites. *N* = 301. Coefficients are standardized HC3-robust composite associations estimated with coded demographic and usage controls. *R*^2^ values are equation-level values. Because the questionnaire blocks lack discriminant separation, the coefficients are descriptive rather than construct-specific effects. Bootstrap coefficient-product results are reported in Supplementary Table S2.

In the attitude equation, PEOU (*β* = 0.470, 95% CI [0.342, 0.599], *p* < 0.001) and PPU (*β* = 0.431, 95% CI [0.294, 0.569], *p* < 0.001) were each positively associated with ATT. The equation explained 77.8% of variance. EP2a and EP2b were consistent with the observed composite associations. The coefficients suggest that both manageable operation and recognized practical value accompany a favorable learning attitude.

ATT (*β* = 0.446, 95% CI [0.342, 0.550], *p* < 0.001) and PPU (*β* = 0.480, 95% CI [0.379, 0.582], *p* < 0.001) were associated with INT. The intention equation explained 78.3% of variance, and the observed associations were consistent with EP3a and EP3b. Practical usefulness had a slightly larger partial coefficient than attitude, consistent with an instrumental route in which career, efficiency, competitiveness, and problem-solving expectations motivate use. The difference between coefficients should not be overinterpreted because their confidence intervals overlap and the constructs are highly correlated.

The continuance equation explained 79.3% of variance. In light of HTMT values ranging from 0.983 to 1.007 and the dominant first component, it primarily reflects shared variance and empirical overlap among the positively worded appraisal scales. INT (*β* = 0.350, 95% CI [0.236, 0.465], *p* < 0.001), ATT (*β* = 0.324, 95% CI [0.202, 0.446], *p* < 0.001), and PPU (*β* = 0.267, 95% CI [0.137, 0.396], *p* < 0.001) each retained a positive association with CONT. The observed associations were consistent with EP4a–EP4c. Focal-predictor VIF values ranged from 3.55 to 4.69, below a conventional value of five but high enough to reinforce caution. The coefficients indicate that continuation is not reducible to stated initial intention: favorable learning orientation and perceived practical value remain associated with the outcome when intention is included.

Figure 2 summarizes the focal path coefficients and equation-level explanatory power.

### 4.5 Supplementary diagnostic analyses

Conventional coefficient-product and configurational analyses were retained only as supplementary diagnostics of whether the highly overlapping questionnaire blocks yielded information beyond the dominant shared-response dimension. Bootstrap coefficient products were positive, whereas fsQCA produced a single high-response conjunction and its low-response mirror image, with no evidence of equifinality or configurational asymmetry. Because these analyses use the same non-discriminant composites identified in the measurement audit, they provide no independent evidence for construct-specific psychological mechanisms. Full estimates and calibration sensitivity results are reported in Supplementary Tables S2, S3, and Supplementary Figure S1.

### 4.6 Theoretical discussion

The principal finding concerns measurement rather than structural relations. Although the five questionnaire composites can be arranged in an acceptance-to-continuance sequence, HTMT, PCA, CFA, and the collapsed-readiness benchmark indicate that respondents did not distinguish these domains sufficiently to support five independent mechanisms. The questionnaire therefore exhibits a broad shared evaluative dimension spanning perceived manageability, practical value, positive learning evaluation, deliberate intention, and willingness to continue; the present data cannot determine whether this common dimension is substantive, method-related, or a combination of both. The regression and bootstrap results describe the distribution of shared variance within this profile and are retained as a reproducible benchmark, not as evidence of temporal, causal, or construct-specific pathways.

The psychological implication is therefore not that TAM has been disproved, but that its usual dimensional decomposition cannot be assumed in AI-related student surveys. A TAM-type explanation requires more than individually reliable subscales: respondents’ answers must also demonstrate that ease, usefulness, attitude, intention, and continuance carry empirically distinguishable information. When this condition fails, interventions directed at a supposedly specific pathway become difficult to interpret because change in one scale may largely track the same global evaluation represented in the others. Future TAM research on AI should therefore treat discriminant validity as a substantive prerequisite for mechanism testing rather than as a routine measurement checkpoint.

General AI-application readiness is defined here as a broad favorable orientation toward learning about and intentionally applying AI, reflected jointly in perceived manageability, practical value, positive learning evaluation, and willingness to continue. It differs from AI literacy, which concerns the knowledge and critical competence required to understand, evaluate, and use AI responsibly, and from digital readiness, which more broadly includes access, infrastructure, digital skills, and willingness to engage with digital technologies. The profile identified here is therefore narrower than digital readiness and less competency-based than AI literacy; it describes perceived orientation rather than demonstrated capability. Because the five blocks are not empirically distinct, the profile should be treated as a descriptive higher-order tendency rather than as a newly validated latent construct.

This distinction has direct implications for AI-literacy education. Ease of operation is not equivalent to usefulness, and neither judgment establishes that a student can evaluate output accuracy, recognize hallucinations, verify sources, protect privacy, disclose AI assistance appropriately, or decide when AI should not be used. If favorable AI appraisals tend to collapse into a global impression, literacy programs should deliberately separate these judgments rather than merely strengthen positive attitudes toward AI. For example, students can be asked to evaluate an AI tool separately for usability, task usefulness, evidential reliability, ethical acceptability, and appropriateness for continued use. Such differentiation would align AI education more closely with critical competence and responsible judgment rather than with adoption enthusiasm alone.

This interpretation also connects with established readiness scholarship. Technology readiness concerns an individual’s propensity to embrace and use new technologies, whereas organizational readiness concerns collective commitment and perceived capability to implement change (Parasuraman and Colby, 2015; Weiner, 2009). The profile observed in the present dataset is narrower and more measurement-bound. It captures a favorable student orientation toward AI application but does not measure the full balance of optimism, innovativeness, discomfort, insecurity, shared commitment, or implementation efficacy found in established readiness frameworks. It should therefore be treated as an empirical readiness profile within this dataset rather than as a replacement for existing individual- or organization-level readiness constructs.

The CFA comparison strengthens this interpretation. Although both models showed apparently excellent absolute fit, the five-factor solution improved chi-square by only 2.059 with ten additional parameters, and its latent correlations ranged from 0.980 to 0.997. The five-factor covariance structure was therefore nearly singular. These findings indicate that excellent fit indices can coexist with construct redundancy and should not be interpreted as confirmation of five distinct psychological mechanisms.

A substantive common orientation is not the only explanation for this covariance pattern. All five blocks were obtained from the same respondents, during the same questionnaire session, using the same 5-point response format, and the focal items are overwhelmingly phrased in the same favorable direction. Such conditions can generate shared variance through acquiescence, halo-like global evaluation, item-context effects, and other common-method or response-style processes (Podsakoff et al., 2003). The present data contain no method-marker variable, alternative measurement method, balanced reverse-keyed block, or repeated measurement occasion capable of separating a substantive general factor from a method factor. Consequently, the observed shared dimension is interpreted descriptively. The term AI-application readiness, where retained, denotes the covariance pattern in these records rather than a newly validated psychological construct.

Within this sample of 301 surveyed students, the observed readiness profile may reflect features of the mainland Chinese platform and higher-education context, including domestic and international systems, uneven institutional access, academic-performance and employability pressures, and concerns about academic integrity. These conditions may increase the practical value attached to AI while also encouraging rapid, efficiency-oriented use. The profile should therefore not be assumed to be culturally universal; comparative research should test whether its composition changes across institutional and national contexts (Fan et al., 2025; Liu et al., 2025; Wang et al., 2024). These contextual interpretations are hypotheses derived from the sample and related literature, not population estimates for all Chinese university students.

A further testable hypothesis concerns cultural variation in the organization of evaluative judgments. Cross-cultural psychology has reported average differences in analytic versus holistic and context-sensitive cognition across Western and East Asian samples (Ji et al., 2000; Varnum et al., 2010). These findings do not establish that the present respondents answered AI items globally because of Chinese culture, and cultural tendencies should not be inferred from this incompletely documented secondary sample. They nevertheless motivate a prospective hypothesis: after measurement invariance and wording effects are controlled, the relative strength of a general AI-evaluation factor versus domain-specific TAM factors may differ across cultural contexts. Cross-cultural studies using balanced item wording, method markers, and matched samples could distinguish a cultural-cognitive explanation from a common-method explanation.

The positive associations of ease and usefulness resemble the findings of Strzelecki (2024), while the relationship between favorable evaluation and continued use is consistent with Yu et al. (2024). The main contribution is therefore not the magnitude of the path coefficients. It is the convergence of CFA, HTMT, PCA, regression, the collapsed benchmark, and fsQCA on the same bounded interpretation: the questionnaire captures a broad readiness orientation more clearly than a sequence of separable mechanisms. Importantly, perceived practical usefulness captures only respondents’ self-reported expectations concerning career relevance, efficiency, competitiveness, and problem solving. It does not demonstrate actual employability, learning gains, policy effectiveness, or institutional performance. Any connection between these perceptions and objective educational, employment, or policy outcomes remains speculative until tested through behavioral, longitudinal, or administrative evidence.

### 4.7 Methodological contribution

The methodological contribution lies in treating discriminant validity as a substantive result. The analysis retained the reliable blocks as descriptive composites, reported bootstrap coefficient products without causal-mediation language, and interpreted the single symmetric fsQCA solution as evidence against equifinality rather than as evidence of multiple configurational pathways.

The study also illustrates the obligations of secondary analysis. Public data support replication, but they do not remove the need to credit data producers, acknowledge prior analyses, disclose incomplete metadata, and respect sampling limits. The public workbook, reconstruction code, and machine-readable outputs provide a reproducible separation between the original data contribution and the present measurement audit and secondary analysis.

### 4.8 Institutional, policy, and competitive-intelligence implications

At the institutional level, AI governance should be connected to student development rather than reduced to prohibited-use rules. Chan’s (2023) ecological policy framework links pedagogical, governance, and operational considerations. The present results add a student-pathway perspective: implementation should be evaluated not only through awareness of rules or platform logins, but also through whether students can operate AI, recognize credible value, verify outputs, disclose use appropriately, and form responsible plans for continued application.

Continuance intention is not an automatic indicator of academic-integrity success. Process visibility can be strengthened through staged submissions, revision or prompt records where appropriate, oral explanation, reflective annotations, and task-specific disclosure (Cotton et al., 2024; Perkins, 2023). Institutional dashboards should therefore combine access and usage indicators with verification performance, responsible-use knowledge, disclosure compliance, student confidence, and selected learning or career outcomes. Because the present data capture perceptions at one time point, they can inform dashboard design but cannot evaluate institutional effectiveness.

A competitive-intelligence interpretation must remain bounded. The dataset contains student perceptions of usability, value, attitude, and intended continuation; it does not measure competitor behavior, institutional competitiveness, labor-market outcomes, or strategic performance. It therefore cannot support claims that AI adoption improves a university’s competitive position. Aggregated, privacy-preserving student data may nevertheless contribute to institutional scanning when combined with curriculum mapping, employer input, platform-risk assessment, and policy monitoring. Such information should be used to prioritize support, not to rank individual students or normalize intrusive surveillance.

For institutions serving student populations and platform environments similar to those represented in the present study, a cautious implication is to avoid dependence on a single vendor because platforms differ in language support, access, cost, data governance, and interoperability. Strategy should emphasize transferable skills—problem framing, output evaluation, source verification, privacy awareness, disclosure, and accountable human decision-making—consistent with UNESCO’s (2023) human-centered guidance.

## 5 Final considerations

This measurement-aware secondary analysis examined perceived ease of use, perceived practical usefulness, learning attitude, intention to use, and continuance intention in 301 records contained in a public dataset described by its repository as university-student responses from mainland China. The five 4-item blocks were individually reliable but showed inadequate discriminant separation, near-unit HTMT values, a dominant first component, near-unit latent-factor correlations, and negligible improvement of the five-factor CFA over the one-factor benchmark. The central finding is therefore measurement-level rather than confirmation of a five-stage acceptance mechanism.

The response matrix is dominated by a shared evaluative dimension that can be summarized descriptively by a collapsed score. However, the present single-method, predominantly co-directional questionnaire cannot establish whether this shared dimension represents a substantive global orientation toward AI, common-method variance, response-style effects, or a combination of these sources. The collapsed score should therefore not be interpreted as a newly validated AI-application readiness construct. HC3 regressions, coefficient-product analyses, and fsQCA results are best understood as descriptive diagnostics of how the shared variance is distributed rather than as evidence of distinct causal, mediating, or configurational mechanisms.

Several limitations are decisive. The design is cross-sectional, self-reported, and based on non-probability secondary data, so temporal ordering and causal inference are not available. Practical impacts are perceived rather than verified through grades, skill assessments, employment outcomes, or institutional indicators. The public workbook lacks a complete codebook for demographic and usage categories; consequently, subgroup analyses cannot be interpreted responsibly, and the coefficients of the coded controls have no defensible substantive meaning. The item blocks share substantial content and method variance, and all cases have the same code on the AI-use screening item. Submission timestamps are compressed into an interval of approximately 13 min. The dataset curators were not contacted regarding the approximately 13 min compression of submission timestamps; therefore, no clarification beyond the public repository materials was available as of manuscript submission. This pattern may reflect batch export, anonymization, synchronized administration, or another processing artifact; it does not by itself establish invalidity, but it weakens provenance verification. Institutional and regional sampling details are also unavailable. Finally, because the dataset is linked to prior work by its authors, the contribution of the present article is limited to transparent reanalysis and methodological critique.

Future research should use longitudinal designs that separate initial evaluation, trial use, sustained behavior, and learning or career outcomes over time. Surveys should reduce item redundancy, combine favorable and critical indicators, and test measurement invariance across disciplines, academic stages, and levels of AI use. Self-report should be supplemented with behavioral traces, task performance, source-verification accuracy, revision histories, and advisor assessments. Configurational studies also require greater diversity in condition profiles to test genuine substitution and equifinality.

Among the 301 surveyed students, higher reported continuance intention was associated with perceiving AI as manageable and practically valuable, holding a positive learning orientation, and expressing deliberate intention to use it. These associations describe the surveyed sample and should not be generalized to all Chinese university students without probability-based or multi-institutional replication. College counselors and student-support services can use these domains to structure broad, non-subject-specific guidance. Increased continuation should, however, be pursued only alongside verification, ethics, authorship, privacy, and independent judgment. The educational value of AI lies not in repeated use alone, but in the quality of the human decisions that organize that use.

## Data Availability

The original contributions presented in the study are included in the article/Supplementary material, further inquiries can be directed to the corresponding author.

## References

[ref1] Baek C. Tate T. Warschauer M. (2024). “ChatGPT seems too good to be true”: college students’ use and perceptions of generative AI. Comput. Educ. Artif. Intell. 7:100294. doi: 10.1016/j.caeai.2024.100294

[ref2] Bai Y. Wang S. (2025). Impact of generative AI interaction and output quality on university students’ learning outcomes: a technology-mediated and motivation-driven approach. Sci. Rep. 15:24054. doi: 10.1038/s41598-025-08697-6, 40617950 PMC12228813

[ref3] Bhattacherjee A. (2001). Understanding information systems continuance: an expectation-confirmation model. MIS Q. 25, 351–370. doi: 10.2307/3250921

[ref4] Bond M. Khosravi H. de Laat M. Bergdahl N. Negrea V. Oxley E. . (2024). A meta systematic review of artificial intelligence in higher education: a call for increased ethics, collaboration, and rigour. Int. J. Educ. Technol. High. Educ. 21:4. doi: 10.1186/s41239-023-00436-z

[ref5] Chan C. K. Y. (2023). A comprehensive AI policy education framework for university teaching and learning. Int. J. Educ. Technol. High. Educ. 20:38. doi: 10.1186/s41239-023-00408-3

[ref6] Chan C. K. Y. Hu W. (2023). Students’ voices on generative AI: perceptions, benefits, and challenges in higher education. Int. J. Educ. Technol. High. Educ. 20:43. doi: 10.1186/s41239-023-00411-8

[ref7] Chen T.-C. Kuo F.-H. Lin M.-M. Li Z.-Y. (2025) A study on the relationship between learning motivation and learning satisfaction from the perspective of AI usage attitude and behavior Proceedings of the 2025 4th International Conference on Artificial Intelligence and Education (ICAIE’25) New York, NY: Association for Computing Machinery

[ref8] Chiu T. K. F. Xia Q. Zhou X. Chai C. S. Cheng M. (2023). Systematic literature review on opportunities, challenges, and future research recommendations of artificial intelligence in education. Comput. Educ. Artif. Intell. 4:100118. doi: 10.1016/j.caeai.2022.100118

[ref9] Cotton D. R. E. Cotton P. A. Shipway J. R. (2024). Chatting and cheating: ensuring academic integrity in the era of ChatGPT. Innov. Educ. Teach. Int. 61, 228–239. doi: 10.1080/14703297.2023.2190148

[ref10] Crompton H. Burke D. (2023). Artificial intelligence in higher education: the state of the field. Int. J. Educ. Technol. High. Educ. 20:22. doi: 10.1186/s41239-023-00392-8

[ref11] Davis F. D. (1989). Perceived usefulness, perceived ease of use, and user acceptance of information technology. MIS Q. 13, 319–340. doi: 10.2307/249008

[ref12] Delcker J. Heil J. Ifenthaler D. Seufert S. Spirgi L. (2024). First-year students’ AI-competence as a predictor for intended and de facto use of AI-tools for supporting learning processes in higher education. Int. J. Educ. Technol. High. Educ. 21:18. doi: 10.1186/s41239-024-00452-7

[ref13] Deng R. Jiang M. Yu X. Lu Y. Liu S. (2025). Does ChatGPT enhance student learning? A systematic review and meta-analysis of experimental studies. Comput. Educ. 227:105224. doi: 10.1016/j.compedu.2024.105224

[ref14] Fan L. Deng K. Liu F. (2025). Educational impacts of generative artificial intelligence on learning and performance of engineering students in China. Sci. Rep. 15:26521. doi: 10.1038/s41598-025-06930-w, 40691450 PMC12280127

[ref15] Farrokhnia M. Banihashem S. K. Noroozi O. Wals A. (2024). A SWOT analysis of ChatGPT: implications for educational practice and research. Innov. Educ. Teach. Int. 61, 460–474. doi: 10.1080/14703297.2023.2195846

[ref16] Fiss P. C. (2011). Building better causal theories: a fuzzy set approach to typologies in organization research. Acad. Manag. J. 54, 393–420. doi: 10.5465/amj.2011.60263120

[ref17] Grassini S. (2023). Shaping the future of education: exploring the potential and consequences of AI and ChatGPT in educational settings. Educ. Sci. 13:692. doi: 10.3390/educsci13070692

[ref18] Hair J. F. Jr. Hult G. T. M. Ringle C. M. Sarstedt M. (2022). A Primer on Partial Least Squares Structural Equation Modeling (PLS-SEM). 3rd Edn Thousand Oaks, CA: SAGE.

[ref19] Henseler J. Ringle C. M. Sarstedt M. (2015). A new criterion for assessing discriminant validity in variance-based structural equation modeling. J. Acad. Mark. Sci. 43, 115–135. doi: 10.1007/s11747-014-0403-8

[ref20] Ji L.-J. Peng K. Nisbett R. E. (2000). Culture, control, and perception of relationships in the environment. J. Pers. Soc. Psychol. 78, 943–955. doi: 10.1037/0022-3514.78.5.943, 10821200

[ref21] Kasneci E. Sessler K. Küchemann S. Bannert M. Dementieva D. Fischer F. . (2023). ChatGPT for good? On opportunities and challenges of large language models for education. Learn. Individ. Differ. 103:102274. doi: 10.1016/j.lindif.2023.102274

[ref22] Kim J. Klopfer M. Grohs J. R. Eldardiry H. Weichert J. Cox L. A. II . (2025). Examining faculty and student perceptions of generative AI in university courses. Innov. High. Educ. 50, 1281–1313. doi: 10.1007/s10755-024-09774-w

[ref23] Kuo F.-H. Chen T.-C. (2025). Factors influencing Chinese university students’ learning attitudes and intentions for continuous use of artificial intelligence courses. Mendeley Data. doi: 10.17632/hx6m853xks.1

[ref24] Liu X. Xiao Y. Li D. (2025). Assessing strategic use of artificial intelligence in self-regulated learning: instrument development and evidence from Chinese university students. Int. J. Educ. Technol. High. Educ. 22:69. doi: 10.1186/s41239-025-00567-5

[ref25] Luo J. Zheng C. Yin J. Teo H. H. (2025). Design and assessment of AI-based learning tools in higher education: a systematic review. Int. J. Educ. Technol. High. Educ. 22:42. doi: 10.1186/s41239-025-00540-2

[ref26] MacKinnon D. P. Lockwood C. M. Williams J. (2004). Confidence limits for the indirect effect: distribution of the product and resampling methods. Multivar. Behav. Res. 39, 99–128. doi: 10.1207/S15327906MBR3901_4, 20157642 PMC2821115

[ref27] MacKinnon J. G. White H. (1985). Some heteroskedasticity-consistent covariance matrix estimators with improved finite sample properties. J. Econom. 29, 305–325. doi: 10.1016/0304-4076(85)90158-7

[ref28] Ouyang F. Zheng L. Jiao P. (2022). Artificial intelligence in online higher education: a systematic review of empirical research from 2011 to 2020. Educ. Inf. Technol. 27, 7893–7925. doi: 10.1007/s10639-022-10925-9

[ref29] Pappas I. O. Woodside A. G. (2021). Fuzzy-set qualitative comparative analysis (fsQCA): guidelines for research practice in information systems and marketing. Int. J. Inf. Manag. 58:102310. doi: 10.1016/j.ijinfomgt.2021.102310

[ref30] Parasuraman A. Colby C. L. (2015). An updated and streamlined technology readiness index: TRI 2.0. J. Serv. Res. 18, 59–74. doi: 10.1177/1094670514539730

[ref31] Perkins M. (2023). Academic integrity considerations of AI large language models in the post-pandemic era: ChatGPT and beyond. J. Univ. Teach. Learn. Pract. 20:7. doi: 10.53761/1.20.02.07

[ref32] Podsakoff P. M. MacKenzie S. B. Lee J.-Y. Podsakoff N. P. (2003). Common method biases in behavioral research: a critical review of the literature and recommended remedies. J. Appl. Psychol. 88, 879–903. doi: 10.1037/0021-9010.88.5.879, 14516251

[ref33] Ragin C. C. (2008). Redesigning Social Inquiry: Fuzzy Sets and Beyond. Chicago, IL: University of Chicago Press.

[ref34] Schneider C. Q. Wagemann C. (2012). Set-Theoretic Methods for the Social Sciences: A Guide to Qualitative Comparative Analysis. Cambridge: Cambridge University Press.

[ref35] Stöhr C. Ou A. W. Malmström H. (2024). Perceptions and usage of AI chatbots among students in higher education across genders, academic levels and fields of study. Comput. Educ. Artif. Intell. 7:100259. doi: 10.1016/j.caeai.2024.100259, 42574925

[ref36] Strzelecki A. (2024). To use or not to use ChatGPT in higher education? A study of students’ acceptance and use of technology. Interact. Learn. Environ. 32, 5142–5155. doi: 10.1080/10494820.2023.2209881

[ref37] Tlili A. Shehata B. Adarkwah M. A. Bozkurt A. Hickey D. T. Huang R. . (2023). What if the devil is my guardian angel: ChatGPT as a case study of using chatbots in education. Smart Learn. Environ. 10:15. doi: 10.1186/s40561-023-00237-x

[ref38] UNESCO (2023). Guidance for Generative AI in Education and Research. Paris: UNESCO.

[ref39] Varnum M. E. W. Grossmann I. Kitayama S. Nisbett R. E. (2010). The origin of cultural differences in cognition: the social orientation hypothesis. Curr. Dir. Psychol. Sci. 19, 9–13. doi: 10.1177/0963721409359301, 20234850 PMC2838233

[ref40] Wang X. Xu X. Zhang Y. Hao S. Jie W. (2024). Exploring the impact of artificial intelligence application in personalized learning environments: thematic analysis of undergraduates’ perceptions in China. Humanit. Soc. Sci. Commun. 11:1644. doi: 10.1057/s41599-024-04168-x

[ref41] Weiner B. J. (2009). A theory of organizational readiness for change. Implement. Sci. 4:67. doi: 10.1186/1748-5908-4-67, 19840381 PMC2770024

[ref42] Yang Y. Luo J. Yang M. Yang R. Chen J. (2024). From surface to deep learning approaches with generative AI in higher education: an analytical framework of student agency. Stud. High. Educ. 49, 817–830. doi: 10.1080/03075079.2024.2327003

[ref43] Yu C. Yan J. Cai N. (2024). ChatGPT in higher education: factors influencing ChatGPT user satisfaction and continued use intention. Front. Educ. 9:1354929. doi: 10.3389/feduc.2024.1354929

[ref44] Zheng H. Qian Y. Wang Z. Wu Y. (2023). Research on the influence of e-learning quality on the intention to continue e-learning: evidence from SEM and fsQCA. Sustainability 15:5557. doi: 10.3390/su15065557

[ref45] Zhou J. Zhang H. (2024). Factors influencing university students’ continuance intentions towards self-directed learning using artificial intelligence tools: insights from structural equation modeling and fuzzy-set qualitative comparative analysis. Appl. Sci. 14:8363. doi: 10.3390/app14188363

[ref46] Zimmerman B. J. (2002). Becoming a self-regulated learner: an overview. Theory Pract. 41, 64–70. doi: 10.1207/S15430421TIP4102_2

