# Supplementary material for: Beyond technology acceptance: AI-application readiness and continuance intention among Chinese university students—a measurement-aware secondary analysis
Source: Front Psychol. 2026 Oct 1;17:1963259. doi: 10.3389/fpsyg.2026.1963259 (PMC13668810; doi:10.3389/fpsyg.2026.1963259)
Supplement: Supplementary file 1 [file Supplementary_file_1.docx]

Supplementary Material

***Beyond Technology Acceptance: Measurement Overlap and Continuance Intention in a Public Dataset of 301 University-Student Responses from Mainland China***

Supplementary Tables S1–S3 and Supplementary Figure S1

This supplementary file reports internal data-provenance checks, bootstrap coefficient-product diagnostics, fsQCA calibration sensitivity, and the supplementary configurational visualization. These materials are provided to document analyses that are secondary to the manuscript’s main psychometric findings.

# S1. Data provenance and internal integrity audit

The audit was conducted directly on the public workbook 0308AI.xlsx used in the manuscript. It evaluates internal file consistency and observable response-pattern properties; it does not independently establish respondent recruitment, institutional coverage, or regional sampling. During revision, the dataset authors were contacted to request clarification of these issues, the category coding, the compressed submission timestamps, and the relationship between the workbook and Chen et al. (2025). No response had been received when the revised materials were finalized. Supplementary Table S1 summarizes the verifiable workbook-level checks.

Supplementary Table S1. Data provenance and internal integrity audit.

| **Audit item** | **Verified finding** |
| --- | --- |
| Analytical workbook | 0308AI.xlsx |
| File checksum | SHA-256: ed86b033b1046d11aade6e148aff4ad7a9df84b99b8211d2df80e1a02fda5005 |
| Respondent records | 301 |
| Workbook worksheets | Sheet1 and 001 |
| Focal-item consistency | The 20 item values in worksheet 001 exactly match the corresponding 20 focal-item columns in Sheet1 for all 301 records. |
| Category codebook | No codebook or lookup worksheet translating all category codes into substantive labels is present. |
| Institution/region fields | No institution identifier or province/region sampling field is present in the workbook. |
| Submission timestamps | 2025-01-27 14:51:09 to 15:04:20; total span = 791 s (13 min 11 s). |
| Unique submission timestamps | 184 among 301 records. |
| Recorded response duration | Mean = 173.4 s; median = 164 s; range = 91–281 s. |
| Exact duplicate 20-item profiles | 0 |
| Complete 20-item straight-line profiles | 0 |
| Longest identical-response run | 8 consecutive focal items. |
| External clarification attempt | Dataset authors were contacted during revision; no response had been received when the revised materials were finalized. |
| Interpretive boundary | These checks establish internal workbook consistency and observable response variation, but they do not independently verify recruitment, institutional/region coverage, category meanings, or the process that generated the compressed timestamps. |

***Note.*** The public repository identifies the dataset as originating from university students in mainland China. The workbook-level audit does not independently verify that population description.

# S2. Bootstrap coefficient-product diagnostics

Supplementary Table S2 reports the 10,000-draw case-resampling bootstrap coefficient products retained as descriptive diagnostics. Because the constituent questionnaire blocks lack discriminant separation, these quantities are not interpreted as causal mediation effects.

Supplementary Table S2. Bootstrap coefficient-product associations based on 10,000 case-resampling draws.

| **Indirect association** | **Estimate** | **Boot SE** | **95% CI** | **p** |
| --- | --- | --- | --- | --- |
| PEOU → PPU → CONT | .218 | .053 | [.118, .324] | < .001 |
| PEOU → ATT → CONT | .152 | .036 | [.087, .230] | < .001 |
| PEOU → PPU → ATT → CONT | .114 | .027 | [.067, .172] | < .001 |
| PEOU → ATT → INT → CONT | .074 | .018 | [.042, .113] | < .001 |
| PEOU → PPU → INT → CONT | .138 | .028 | [.085, .195] | < .001 |
| PEOU → PPU → ATT → INT → CONT | .055 | .013 | [.031, .084] | < .001 |
| PPU → ATT → CONT | .140 | .033 | [.082, .209] | < .001 |
| PPU → INT → CONT | .168 | .034 | [.104, .239] | < .001 |
| PPU → ATT → INT → CONT | .067 | .017 | [.038, .104] | < .001 |
| ATT → INT → CONT | .156 | .030 | [.100, .220] | < .001 |
| PEOU total indirect | .751 | .030 | [.692, .812] | < .001 |
| PPU total indirect | .375 | .051 | [.280, .478] | < .001 |

***Note.*** Intervals are percentile bootstrap confidence intervals. These cross-sectional coefficient products are descriptive and should not be interpreted as causal indirect effects.

# S3. fsQCA calibration sensitivity

The primary calibration used 1.5, 3.0, and 4.5 as full non-membership, crossover, and full membership. A sensitivity analysis repeated the calibration using the scale endpoints 1.0 and 5.0 with the same 3.0 crossover. Supplementary Table S3 shows that the mirror-image high/low configuration pattern was unchanged.

Supplementary Table S3. fsQCA calibration sensitivity.

| **Calibration** | **Outcome** | **Configuration** | **Consistency** | **PRI** | **Raw coverage** |
| --- | --- | --- | --- | --- | --- |
| 1.5 / 3.0 / 4.5 | High CONT | PPU×PEOU×ATT×INT | .961 | .947 | .789 |
| 1.5 / 3.0 / 4.5 | Low CONT | ~PPU×~PEOU×~ATT×~INT | .958 | .943 | .708 |
| 1.0 / 3.0 / 5.0 | High CONT | PPU×PEOU×ATT×INT | .965 | .944 | .796 |
| 1.0 / 3.0 / 5.0 | Low CONT | ~PPU×~PEOU×~ATT×~INT | .959 | .930 | .720 |

***Note.*** N = 301. The alternative calibration changes fuzzy-set membership anchors while retaining the 3.0 crossover. The substantive mirror-image high/low interpretation is unchanged.

# S4. Supplementary configurational visualization

Supplementary Figure S1 visualizes the primary fsQCA solutions. The high-CONT conjunction and low-CONT mirror image reinforce the absence of equifinality or configurational asymmetry in these data; given the severe measurement overlap among conditions, the figure is treated as a descriptive diagnostic rather than evidence of distinct configurational mechanisms.

Supplementary Figure S1. fsQCA diagnostic for high and low continuance intention under the primary calibration.

***Note.*** N = 301. Primary calibration anchors were 1.5, 3.0, and 4.5. High CONT: consistency = .961, PRI = .947, raw coverage = .789. Low CONT: consistency = .958, PRI = .943, raw coverage = .708. The mirror-image solutions indicate no configurational asymmetry.
